# Network-enabled gene expression analysis

**DOI:** 10.1186/1471-2105-13-167

**Published:** 2012-07-16

**Authors:** David Edwards, Lei Wang, Peter Sørensen

**Affiliations:** 1 Department of Molecular Biology and Genetics, Aarhus University, Blichers Allé 20, 8830 Tjele, Denmark

## Abstract

**Background:**

Although genome-scale expression experiments are performed routinely in biomedical research, methods of analysis remain simplistic and their interpretation challenging. The conventional approach is to compare the expression of each gene, one at a time, between treatment groups. This implicitly treats the gene expression levels as independent, but they are in fact highly interdependent, and exploiting this enables substantial power gains to be realized.

**Results:**

We assume that information on the dependence structure between the expression levels of a set of genes is available in the form of a Bayesian network (directed acyclic graph), derived from external resources. We show how to analyze gene expression data conditional on this network. Genes whose expression is directly affected by treatment may be identified using tests for the independence of each gene and treatment, conditional on the parents of the gene in the network. We apply this approach to two datasets: one from a hepatotoxicity study in rats using a PPAR pathway, and the other from a study of the effects of smoking on the epithelial transcriptome, using a global transcription factor network.

**Conclusions:**

The proposed method is straightforward, simple to implement, gives rise to substantial power gains, and may assist in relating the experimental results to the underlying biology.

## Background

Although genome-scale expression experiments are performed routinely in biomedical research, understanding the data they generate remains a major challenge. A widely used approach to relate such data to biology is *gene set enrichment analysis*[1]. This shifts focus from the regulation of individual genes to the regulation of gene sets, i.e. pre-defined sets of genes that share a common biological function or feature such as chromosomal location. One motivation for the approach is that if genes in a set are closely related, they can be expected to have similar expression patterns, and exploiting this may increase power. Another is that analysis at the level of individual genes may often be difficult to interpret biologically, whereas higher-level analyses hopefully allow a more coherent picture of the involved biological processes to emerge. Many methods of gene set enrichment analysis have been proposed (see [2] for a useful survey) and there exists a burgeoning literature on the topic. Very commonly, gene sets are defined on the basis on gene ontology [3] or pathway membership [4], using resources such as the Kyoto Encyclopedia of Genes and Genomes [5].

Recently there has been intense interest in methods that build on the information in biological networks, that is to say, methods that exploit the topology rather than just the set of genes in the network. We briefly summarize some of the methods proposed.

One approach extends gene set enrichment analysis by defining scores that build on network topology. For example, gene set scores that can be expressed as sums of pairwise weights between genes in the set may be modified by weighting gene pairs by the inverse of their path distance in the network [6]. Significance tests using the scores are then constructed, to assess whether the pathway is regulated. Similarly, scores that take the proximity of differentially expressed genes in the pathway into account have been proposed [7,8].

Another approach makes explicit use of network models for the expression data. In [9], fold changes are discretized into two levels: differentially expressed or equally expressed, and a Markov random field model (an undirected graphical model) for the fold changes whose parametrization utilizes pathway topology is adopted. Bayesian inference is used in which prior distributions for the parameters are assumed and posterior distributions derived using Gibbs sampling. These are used to identify differentially expressed genes. A similar approach has recently been proposed to relate genes/markers to disease for genome-wide association studies [10]. As an alternative to Gibbs sampling, which is computationally intense, the Bayesian score criterion [11] can be used as an approximation to the posterior probability of the network, and the significance of the pathway may be assessed on the basis of a randomization test of the score [12].

An alternative network-based approach [13] represents a network as a directed acyclic graph to which a latent variable (or random effect) for each gene representing its baseline level is added, allowing a mixed effect model for the expression levels to be derived. This allows inference to be based on linear mixed effect model techniques. For example, tests of treatment effect on a node set can be derived as Wald tests, making use of standard approximation methods.

Methods exploiting pathway structure have also been proposed for other, related purposes. For classification, knowledge of an undirected gene network has been used to develop classifiers of gene profiles by performing a spectral decomposition of the expression profiles with respect to the eigenfunctions of the graph [14]. For prediction, a modified Lasso algorithm using a penalty function that takes pathway structure into account has been proposed [15]. Similarly, a modified boosting algorithm which incorporates the pathway structure has been proposed [16].

In the following section we describe a simple way to incorporate a known network or pathway into the analysis of gene expression data. This entails augmenting the network with a discrete node, representing the treatment or class variable. We show that this leads to a simple modification of conventional differential expression analysis. The augmented network contains a discrete as well as multiple continuous (Gaussian) nodes: networks containing both types of node are usually called hybrid networks. With a few recent exceptions [17-20], hybrid networks have been little used in network biology. They may be useful, for example, when modeling expression data from comparative experiments (with discrete design variables) or when seeking to integrate such data into a wider biological context (with discrete genotypic or phenotypic information). Since hybrid networks may be unfamiliar to some readers we describe the framework carefully in the next section, but stress that no novel theory is involved.

## Methods

### The model framework

We suppose that data from a gene expression study are available, in the form of an *N *×* p* data matrix *X *= {*x*_*g *_:* g *∈* V*}, where *N* is the number of observations and *V* is a set of *p* genes, together with an *N*×1 vector *T* containing the treatment group information. The key assumption we make is that the steady state (or unperturbed) distribution of the gene expression levels follows a given Bayesian network model, specified in the form of a DAG (directed acyclic graph) $G=\left(V,,,E\right)$, where *E* is a set of directed edges between the nodes in *V*. This network is assumed to have been obtained from external sources, as we illustrate below.

Under such a model, the joint distribution of the data *X *= {*x*_*g *_:* g *∈* V*} factorizes into a product of conditional distributions

(1) $$f(X)=\underset{g\in V}{\prod }f\left(x_{g}\;|\;x_{{\text{pa}}_{G}(g)}\right)$$

where ${\text{pa}}_{G}\left(g\right)$ is the parents of node *g* ∈ *V* in G, that is, the set of nodes with an arrow to *g* in G. One of the strengths of Bayesian network methodology is the freedom to use any univariate models for the conditional distributions. In this paper we assume Gaussian models, in which the conditional distributions are linear regressions of *x*_*g*_ with covariates given by the variables $x_{{\text{pa}}_{G}(g)}$: the Gaussian assumption is not critical for the approach, as we further discuss below.

Another strength of the methodology is the ability to read from the graph which conditional independences hold under the model, using the property of d-separation [21,22]. For disjoint variable sets *U*, *V*, W ⊆ *V*we write

$$x_{U}\;╨\;x_{V}|x_{W}$$

to mean that *x*_*U*_ and *x*_*V*_ are conditionally independent given *x*_*W*_.

To model the effect of the treatment or class variable *T*, we add a node labeled *T* to *V*, and suppose that the treatment directly affects a set of genes *V*_*T*_⊆ *V*. Thus we consider DAGs of the form

$$G(E)=(V\cup \{T\},E\cup E_{T})$$

where *E*_*T*_ is a set of additional edges of the form (*T*,*g*) for *g*∈*V*_*T*_. We suppose that the object of the analysis is to find (i.e., estimate) *V*_*T*_.

We assume that $G\left(E\right)$ defines a Bayesian network model for (*XT*). Since $G\left(E\right)$ contains both discrete and Gaussian nodes, it is a hybrid Bayesian network [23]. From (1) we have that

(2) $$\begin{array}{lll} f(X^{\prime \prime }) & =\underset{y\in V\cup \{T\}}{\prod }f\left(x_{y}\;|\;x_{{\text{pa}}_{G(E)}(g)}\right)\; & \;\\ & =f(x_{T})\left[\underset{g\in V}{\prod }f(x_{g}\;|\;x_{{\text{pa}}_{G(E)}(g)})\right]\; \end{array}$$

Comparing (1) with (2) we see two changes: firstly, a term *f*(*x*_*T*_)=Pr(*x*_*T*_) is introduced. Since we usually condition on *T* this term is of little interest. Secondly, for those genes *g* in *V*_*T*_ we need to let the conditional distribution of *x*_*g*_depend on *x*_*T*_ as well as $x_{{\text{pa}}_{G}(g)}$. The simplest way to do this is to include an additive treatment effect term, corresponding to a simple shift in the conditional distributions, so that the coefficients of the remaining covariates $x_{{\text{pa}}_{G}(g)}$ do not depend on the treatment. This implies that the treatment may affect the mean gene expression values but not their covariances. In some applications it may be more appropriate to let the coefficients vary by treatment, by specifying treatment by covariate interactions, but in the following we assume additive treatment effects.

Maximum likelihood estimates under the model (2) can be obtained by maximizing the likelihood for each factor separately: since these are all standard models, this is easily done. The likelihood ratio test (or *deviance*) statistic for testing $G\left(E_{0}\right)$ under $G\left(E_{1}\right)$ when $E_{0}\subseteq E_{1}$ is $2({\hat{l}}_{0}-{\hat{l}}_{1})$, where ${\hat{l}}_{0}$ and ${\hat{l}}_{1}$ are the maximized log-likelihood values under $G\left(E_{0}\right)$ and $G\left(E_{1}\right)$. Under $G\left(E_{0}\right)$ the deviance has an asymptotic $\chi_{f}^{2}$ distribution where *f * is the difference in the number of parameters of the two models.

An important special case occurs when *E*_0_and *E*_1_ differ by one edge only, say *T* →g. Observe that in (2), removing (*Tg*) only affects the component $f(x_{g}\;|\;x_{{\text{pa}}_{G(E)}(g)})$. Thus a test for the removal of the edge *T* → *g* from $G\left(E_{1}\right)$ is a test of

(3) $$x_{g}\;╨\;x_{T}|x_{S}\;\text{versus}\;x_{g}╨x_{T}|x_{S}$$

where $S={\text{pa}}_{G}\left(g\right)$. That is, it is a test of the hypothesis, say $H_{g}$, that *x*_*g*_ is conditionally independent of *x*_*T*_ given the parents of *g* in G. It is natural to evaluate such a test in the conditional distribution of *x*_*g*_ given $x_{S\cup \{T\}}$[22, p.110-2]. This follows a linear regression model for *x*_*g*_with covariates *x*_*S*_ and a discrete term for *x*_*T*_, and so to test $H_{g}$ we test whether the coefficient(s) of the discrete term are zero in this model. This is a classical F-test (or when *T* is binary, a t-test) which is valid in small samples under the usual distributional assumptions. Since it does not depend on which other edges are in *E*_1_, we can associate each edge *T* →*g*with an F-test. This is in contrast to, for example, variable selection in linear models where evidence for the presence or absence of a term depends on which other terms are taken to be present in the model.

**Figure 1:**
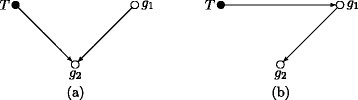
**Comparison of marginal and conditional tests.** Comparison of conditional and marginal tests for two models. Under (**a**), where *T* has a direct effect on *g*_2_, *T* ⊥̸ ⊥*g*_2_and *T* ⊥̸ ⊥*g*_2_ | *g*_1_, but the conditional test will generally have greater power than the marginal test, since using *g*_1_as a regressor will explain some proportion of *g*_2_’s variation. Under (**b**), where *T* does not have a direct effect on *g*_2_, *T* ⊥̸ ⊥*g*_2_but *T* ╨ *g*_2_ | *g*_1_. Hence the conditional null hypothesis holds, and the Type II error of the conditional test is less than *α*, the Type I error of the test. In contrast, the marginal hypothesis is more likely to be rejected, and the Type II error is inflated.

Testing the conditional independence of each gene and treatment, given the parents of the gene in the network can be regarded as a simple modification of conventional methods for differential expression analysis that are based on tests of marginal independence between treatment and genes. To compare and contrast the conditional and marginal approaches, consider the two models relating a treatment *T* to the expression levels of two genes, *g*_1_ and *g*_2_ shown in Figure 1. Conditioning increases power when there is a direct treatment effect, and reduces type II error when there is an indirect but no direct effect, so making the inference more precise.

Note also that the marginal approach can be regarded as the special case of the conditional approach that occurs when G=(V,∅), so that ${\text{pa}}_{G}(g)=\emptyset$ for all *g*∈*V*.

Since multiple hypotheses are tested, use of conventional significance level thresholds would inflate the false positive rate. Many approaches to correct for multiplicity are available [24]. When the number of genes in the network is large, control of the false discovery rate may be desirable. However, when the number of genes is small, most methods for control of false discovery rate perform badly [25], and other methods have been suggested [26,27]. One option is to use a classical multiple test procedure such as Holm’s step-down procedure [28], as we illustrate in the next section.

## Results

In this section we describe two applications of the method.

### Hepatotoxicity and the PPAR pathway

Here we describe the analysis of data taken from a hepatotoxicity study in rats (*Rattus norvegicus*) available from the Gene Expression Omnibus [29], a public data repository under the auspices of the National Center for Biological Information. The study has ID GSE24363.

The stated objective of the study was to use microarray gene expression data acquired from the liver of rats exposed to hepatotoxicants to build classifiers for prediction of liver necrosis. In the study 418 rats were exposed to one of eight compounds (1,2-dichlorobenzene, 1,4-dichlorobenzene, bromobenzene, monocrotaline, N-nitrosomorpholine, thioacetamide, galactosamine, and diquat dibromide). All eight compounds were studied using standardized procedures, i.e. a common array platform (Affymetrix Rat 230 2.0 microarray), experimental procedures and data retrieving and analysis processes. For each compound, four to six male, 12 week old F344 rats were exposed to a zero dose, low dose, mid dose(s) or a high dose of the toxicant and sacrificed at 6, 24 or 48 hrs later. At necropsy, liver was harvested for RNA extraction, histopathology, and clinical chemistry assessments.

For simplicity we use the subset of data from the study pertaining to 1,2-dichlorobenzene, and compare active with control treatments (ignoring the effects of dose and exposure time). The preprocessing steps are described on the GEO website. In all there were 46 arrays in the subset: 12 animals were in the control group, and 34 animals were exposed to active drug.

Peroxisome proliferator-activated receptors (PPARs) are a group of nuclear receptor proteins that function as transcription factors, playing essential roles in the regulation of cellular differentiation, development, and metabolism of higher organisms. Several types have been identified, denoted PPAR-*α*, PPAR-*δ* and PPAR-*γ*. The signalling pathway controlling the expression of the PPARs and their targets has been well-studied. We are interested in studying the effects of the hepatotoxicant on this pathway.

We obtained a copy of the KEGG [5] PPAR signalling pathway for *Rattus norvegicus* using the SPIA package [30]. All edges correspond to transcription factor/ target gene relations. Excluding genes that are not present on the array, we obtain a DAG with 78 nodes and 287 edges, which is used in the analysis.

To examine the effect of treatment on the network, the network-based tests of $x_{T}\;⊥\;\;\;⊥\;x_{g}|x_{\text{pa}(g)}$ for each gene *g* were compared to marginal t-tests, that is, of *x*_*T*_ ╨ *x*_*g*_. In both cases, to correct for multiplicity we use Holm’s step-down procedure [28] to obtain simultaneous (i.e., multiplicity-adjusted) *p*-values, denoted $\left\{\tilde{p_{g}},g\in V\right\}$. If we reject precisely those hypotheses $H_{g}$ for which ${\tilde{p}}_{g}<\alpha$, the family-wise error rate is controlled, i.e. the probability that this procedure results in *any* false rejections is less or equal to *α*. This is true irrespective of which hypotheses in $H=\{H_{g},g\in V\}$ are true or false. Here false rejections equate to false edge inclusions. This is a cautious approach, that aims to identify the edges which are certainly present in *E*: see [31].

**Figure 2:**
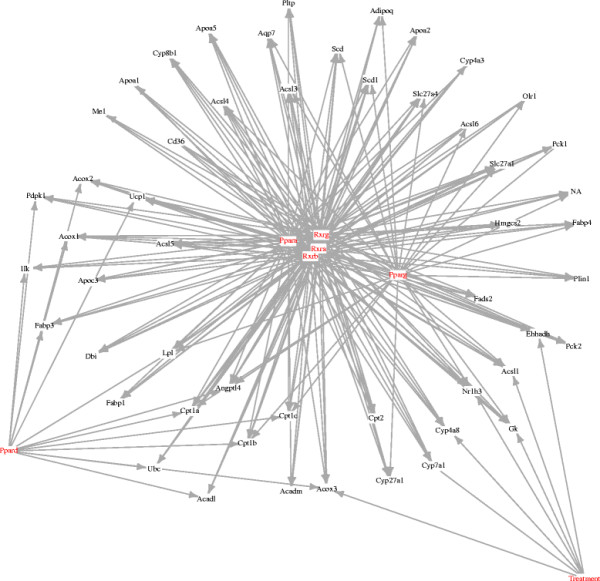
**Augmented PPAR pathway.** An inferred PPAR pathway showing the effects of treatment (multiplicity-adjusted p-values less than 0.05). Transcription factors are shown in red.

Using the network-based tests, $\tilde{p}<0.05$ for seven genes, but none using the marginal tests. Figure 2 shows the augmented PPAR pathway showing the seven genes affected by treatment. Using the same criteria, the marginal analysis detects no treatment effects. Thus in this example, conditioning on the parents of each gene in the network results in a substantial increase in power.

### The effects of smoking

Here we describe the analysis of data taken from a study of the effects of cigarette smoke on the human oral mucosal transcriptome [32]. These are available from the Gene Expression Omnibus [29]: the study has ID GSE17913. In all 40 current smokers and 40 age- and gender- matched never smokers participated in the study, the primary objective of which was to determine the effects of smoking on gene expression in oral epithelial cells. One subject was excluded from the analysis due to poor data quality. For further information on the study and data preprocessing we refer to [32] and [29].

Here we use the data to characterize the effect of smoking on gene expression, making use of a global transcription network constructed using information on human transcription factors (TFs) and their putative target genes (TGs) obtained from the TRANSFAC database [33]. Using the GSEABase package [34] we downloaded data on 95919 (TF,TG) pairs for in all 615 putative TFs and 8945 putative TGs. Of these, the expression levels of 7932 genes were recorded in the GSE17913 study. The (TF,TG) pairs for these genes generate a directed graph, say $G_{0}$, with 7932 nodes (of which 191 are TFs), and 61982 edges. Since some TFs are targets of other TFs, $G_{0}$ contains cycles. The subgraph $G_{1}$ of $G_{0}$ induced by the TFs has 191 nodes and 2711 edges. We derived an acyclic subgraph of $G_{1}$ by omitting edges with the least strength of evidence, in the following way. To derive a measure for the strength of evidence for each edge in $G_{1}$, we fit a linear regression model for the expression of each gene, including its parents in the network as covariates, and use standard significance tests for zero coefficients as a measure of strength of evidence. Starting with the null graph we added edges, ordered in terms of decreasing strength of evidence (increasing *p*-value), that do not generate cycles. This resulted in an acyclic subgraph of $G_{1}$, say $G_{2}$, with 191 nodes and 2110 edges. We then added the nodes and edges in $G_{0}$ but not in $G_{1}$ to $G_{2}$, resulting in a DAG, say G, with 7932 nodes and 61381 edges.

**Figure 3:**
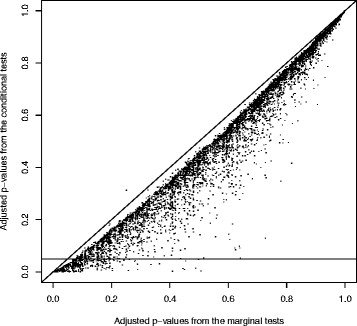
**Comparison of marginal and conditional adjusted p-values.** A scatterplot of multiplicity-adjusted *p*-values obtained using the conditional and tests for the global transcription network.

We applied the method to G, correcting for multiplicity using the method of [35]. Figure 3 compares the adjusted *p*-values from the analysis with the corresponding adjusted values from the marginal analysis not exploiting the network topology. Clearly the conditional tests have substantially greater power. The numbers of genes satisfying the false discovery rate at 1%, 2.5% and 5% were 123, 254 and 478 respectively. In comparison, for a marginal analysis the corresponding numbers were 25, 60 and 162.

**Figure 4:**
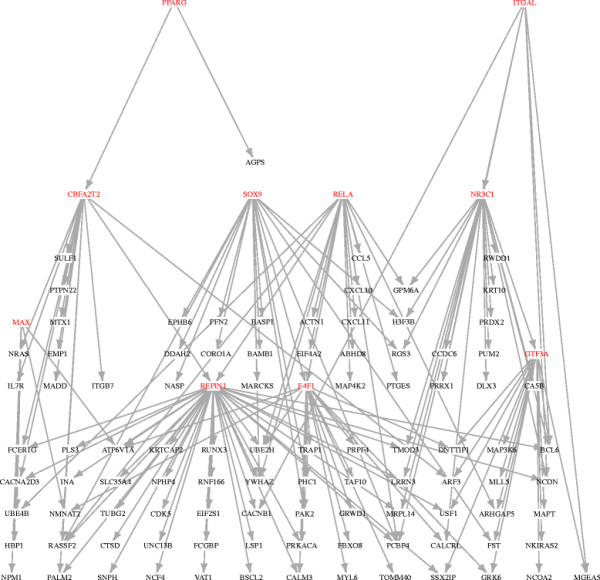
**A subnetwork of the global transcription network.** A subnetwork of the global transcription network. The expression of all genes in the subnetwork are affected by smoking (false discovery rate of 2.5%). Transcription factors are shown in red.

It is instructive to relate the differentially expressed genes to the network topology. For purposes of illustration we consider the 254 genes that satisfy the false discovery rate at 2.5%, of which 10 are transcription factors. The subnetwork of G induced by these 254 genes has one connected component with 107 genes, the remainder being isolated nodes. Figure 4 displays the connected subnetwork, which contains all 10 transcription factors. Thus the analysis has identified a highly connected transcriptional module that is strongly affected by smoking.

## Discussion

We have described a simple way to exploit network information in the analysis of gene expression data, using tests for the conditional independence of each gene and treatment given the parents of the gene in the network. This method can be regarded as an extension of conventional methods of gene expression analysis, that takes the network structure into account. We demonstrated using two examples that the method can result in a substantial increase in power.

In a related approach to the analysis of genetics of gene expression data [36], when relating a marker to the expression of a gene, all expression levels were considered as potential regressors; model selection methods were used to choose the regressors for each marker-gene pair. This is in contrast to the method proposed here, in which an external biological network determines the regressors. We remark that if the regressors for a gene under study include some that are causally downstream of the gene, this may dilute or remove evidence of a direct marker effect on the expression of the gene.

As described above, some authors [9,12] incorporate a dichotomous treatment into a network by letting the network nodes represent discretized fold changes rather than expression levels. We prefer our approach for several reasons. Firstly, the use of fold changes does not appear to be biologically motivated. For example, as we discuss below, it is expected that the mRNA abundance of a transcription factor affects the mRNA abundance of its target genes, but there seems no reason for this to be true for the corresponding fold changes. Secondly, our framework allows direct and indirect treatment effects to be distinguished, and thirdly, it may easily be extended to handle multiple treatment variables, although we do not consider such extensions here.

**Figure 5:**
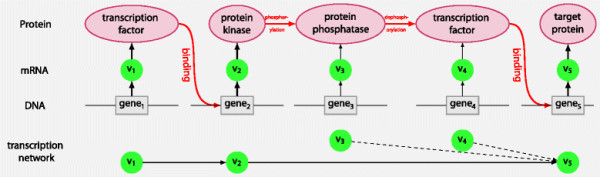
**A hypothetical signalling pathway.** The figure shows a hypothetical signaling pathway adapted from [41], containing two transcription factors, a protein kinase and a protein phosphatase. Three levels are shown: DNA, mRNA and proteins, as well the expected transcription network. At the transcript level, gene 1 affects gene 2 which affects gene 5, but gene 2 does not affect genes 3 or 4. If proteins 3 and/or 4 are rate-limiting, this will be reflected in the dependences shown as dotted arrows.

The approach builds on some assumptions that may or may not be unwarranted. The key assumption is that the steady state distribution of the gene expression levels follows a given Bayesian network. Gene regulation is extremely complex and as yet imperfectly understood, so such an assumption can at best be tentative. We have illustrated the approach using two networks, one based on a signalling pathway and the other constructed using transcription factor/target gene data. Biochemical pathways represent phenomena occurring at the protein level, which are not necessarily reflected at the transcript level: see Figure 5. Thus caution is required when using such pathways as models for gene expression data.

As we have described the method, it assumes that the expression data are Gaussian distributed, but this is not critical. Expression data from microarrays are typically taken to be Gaussian after log transformation [37,38]. Other technologies such as RNA-seq generate discrete count data that are better modeled using Poisson or negative binomial distributions [39]. This can be handled in the current framework by using the appropriate univariate conditional models in (2). What is essential in our context is the availability of an appropriate test for (3), that is, for the conditional independence of treatment and the level of expression of a gene, given the expression levels of its parents in the network. If the correct form of the conditional distribution is in doubt, a permutation test may be used.

An assumption, implicit in both the marginal and the network-based analyses, is that the treatment variable is causal rather than reactive in respect to the gene expression data. Ideally the treatment should represent a randomized intervention, allowing secure causal interpretations, but gene expression studies are rarely randomized. In poorly designed studies, treatment allocation may be confounded with other factors [40]. When this is true, the interpretation of edges *T * → * g*as representing effects of treatment is compromised. The hepatotoxicity study described above is an example of a well-designed study in which treatment represents an non-randomized intervention that is orthogonal to other sources of variation. It is reasonable to interpret treatment effects as causal in such studies. In observational studies, such as the study of the effects of smoking described above, allocation to treatment group (i.e., the process leading to an individual becoming a smoker or non-smoker) could in principle be determined by factors that also influence gene expression, so jeopardizing the interpretation of edges *T *→ * g*as treatment effects. In the actual study, however, it is natural to interpret edges in terms of the effects of tobacco smoke on epithelial cells.

Similar remarks apply to the use of the terms *direct effects* and *indirect effects*: a causal interpretation of these may be unwarranted.

Finally, it is assumed that the treatment affects the parameters of the network but not its topology. In some applications this may not be appropriate. For example, interventions affecting chromatin structure may alter the accessibility of DNA binding sites and hence patterns of regulatory control due to transcription factors.

## Conclusions

A straightforward way to exploit network information in the analysis of gene expression data is to assume that the network models the steady state distribution of the gene expression levels, and that the treatment affects the parameters but not the topology of the network. In this framework, genes whose expression is directly affected by the treatment may be identified using tests for the conditional independence of each gene and treatment given the parents of the gene in the network. This method can be regarded as an extension of conventional methods of gene expression analysis that takes network structure into account. It is simple to implement, gives rise to substantial power gains, and may give insight into the biological processes involved.

## Competing interests

The authors declare that they have no competing interests.

## Author’s contributions

DE conceived the methods, performed the analyses and drafted the manuscript. LW participated in the analyses. All authors read and approved the final manuscript.
